# 1947. Evaluating the Relationship Between Obesity and the Humoral Immune Response to COVID-19 Vaccines

**DOI:** 10.1093/ofid/ofac492.1574

**Published:** 2022-12-15

**Authors:** LaKesha Tables, Fatima Ali, Eric Gaines, Yul Eum Sim, Erica L Johnson, Lilly Immergluck

**Affiliations:** Morehouse School of Medicine, Atlanta, Georgia; Morehouse School of Medicine, Atlanta, Georgia; Morehouse School of Medicine, Atlanta, Georgia; Morehouse School of Medicine, Atlanta, Georgia; Morehouse School of Medicine, Atlanta, Georgia; Morehouse School of Medicine, Atlanta, Georgia

## Abstract

**Background:**

The pro-inflammatory state associated with obesity leads to B- and T-lymphocyte dysfunction that may lead to an inadequate immune response to natural infection and vaccination. Preliminary studies, conducted outside of the US, involving multiple COVID-19 vaccines indicate that obesity may impact antibody response. The objective of this study was to evaluate the role of inflammatory status as a mediator in the relationship between obesity and COVID-19 vaccine immune response in a predominantly African-American population.

**Methods:**

This cross-sectional analysis involved 54 participants ≧ 18 years of age who had completed the primary dosing schedule and booster for Novavax’s recombinant COVID-19 vaccine, NVX-CoV2373. Weight, height, and waist circumference (WC) measurements were taken. Medical history including COVID-19 vaccination and known COVID-19 infection were obtained. Blood samples were taken for measurement of c-reactive protein (CRP) and anti-SARS-CoV-2 spike protein IgG levels. Spearman correlation coefficient was used to assess the presence of a relationship between BMI and CRP, WC and CRP, CRP and spike protein IgG, BMI and spike protein IgG, and finally, WC and spike protein IgG. Mediation analysis was used to evaluate the moderating effect of plasma CRP on the relationship between WC and spike protein IgG while adjusting for suspected confounders. Statistical significance was defined as p < .05.

**Results:**

There was an expected positive relationship between WC and CRP, (ρ = 0.37, p< .05). CRP and spike protein IgG trended towards a weak, negative relationship (ρ= -0.13, p > .05). WC and BMI both trended towards a positive relationship with anti-SARS-CoV-2 spike protein IgG (ρ = 0.29 and 0.15, respectively, p >.05). The mediation analysis showed that WC positively influenced spike protein IgG (p< .05), and this effect was not mediated by CRP.

**Conclusion:**

Inflammation may be negatively associated with antibody response to COVID-19 vaccines. WC and antibody response may be positively related in NVX-CoV2373 recipients, in spite of chronic low-grade inflammation. Further research is needed to fully characterize the impact of obesity on COVID-19 vaccine immunogenic responses.

**Disclosures:**

**Lilly Immergluck, MD, MS**, GSK: Clinical Trial- PI|Merck: Vaccine Trial Site- serve as PI|Moderna: Board Member|Novavax: Part of CoVID-19 Phase 3 Trial through US Covid Prevention Network.

